# Harmonic-Recycling Passive RF Energy Harvester with Integrated Power Management

**DOI:** 10.3390/mi16091053

**Published:** 2025-09-15

**Authors:** Ruijiao Li, Yuquan Hu, Hui Li, Haiyan Jin, Dan Liao

**Affiliations:** 1 School of Information and Communication Engneering, University of Electronic Science and Technology of China, Chengdu 610041, China; lrj@std.uestc.edu.cn (R.L.); yuquan@std.uestc.edu.cn (Y.H.); uestcjhy@163.com (H.J.); liaodan@uestc.edu.cn (D.L.); 2 Jiangxi Communication Terminal Industry Technology Research Institute Co., Ltd., Ji’an 343000, China; 3 Jiangxi Xingtai Technology Co., Ltd., Ji’an 343000, China; 4 Guangdong Electronic Information Engineering Research Institute Shanwei Branch of UESTC, Shanwei 516600, China

**Keywords:** RF energy harvesting, passive rectifier, harmonic energy recycling, VMR, power management, IoT

## Abstract

The rapid growth of low-power Internet of Things (IoT) applications has created an urgent demand for compact, battery-free power solutions. However, most existing RF energy harvesters rely on active rectifiers, multi-phase topologies, or complex tuning networks, which increase circuit complexity and static power overhead while struggling to maintain high efficiency under microwatt-level inputs. To address this challenge, this work proposes a harmonic-recycling, passive, RF-energy-harvesting system with integrated power management (HR-P-RFEH). The system adopts a planar microstrip architecture compatible with MEMS fabrication, integrating a dual-stage voltage multiplier rectifier (VMR) and a stub-based harmonic suppression–recycling network. The design was verified through combined electromagnetic/circuit co-simulations, PCB prototyping, and experimental measurements. Operating at 915 MHz under a 0 dBm input and a 2 kΩ load, the HR-P-RFEH achieves a stable 1.4 V DC output and a peak rectification efficiency of 70.7%. Compared with a conventional single-stage rectifier, it improves the output voltage by 22.5% and the efficiency by 16.4%. The rectified power is further regulated by a BQ25570-based unit to provide a stable 3.3 V supply buffered by a 47 mF supercapacitor, ensuring continuous operation under intermittent RF input. In comparison with the state of the art, the proposed fully passive, harmonic-recycling design achieves competitive efficiency without active bias or adaptive tuning while remaining MEMS- and LTCC-ready. These results highlight HR-P-RFEH as a scalable and fabrication-friendly building block for next-generation energy-autonomous IoT and MEMS systems.

## 1. Introduction

With the rapid advancement of the Internet of Things (IoT), the demand for long-term, stable, and self-powered operation of massive wireless sensor nodes has become increasingly urgent [1]. However, traditional battery-based power supplies suffer from large volume, limited lifespan, and high maintenance costs, which significantly hinder large-scale deployment and sustainable operation of IoT devices [2,3]. As a result, energy harvesting (EH) has emerged as a promising alternative to replace batteries in IoT [4,5,6].

Among various ambient energy sources, Radio Frequency (RF) signals are particularly attractive due to their widespread availability and ease of access, making them a strong candidate for enabling energy-autonomous, low-power IoT devices [7]. Nevertheless, the inherently low power density and fluctuating input power of RF sources pose significant challenges to achieving efficient rectification and power management—especially under microwatt-level conditions, where conventional rectifier structures often fail to maintain efficient energy conversion and stable DC output [8,9].

Recent advancements in microfabrication and Micro-Electro-Mechanical System (MEMS) technologies have further expanded the potential of RF-energy-harvesting devices. Planar microstrip-based layouts, such as the one adopted in this work, are inherently compatible with standard MEMS-compatible fabrication processes, enabling seamless integration into miniaturized wireless sensing platforms. This compatibility allows for the proposed architecture to be manufactured using established photolithography and thin-film deposition techniques, ensuring scalability and reproducibility for the large-scale production of compact, high-performance energy-harvesting modules.

In recent years, adaptive energy management techniques have been developed for piezoelectric, thermoelectric, and photovoltaic harvesting systems. For instance, a hybrid piezo-electromagnetic harvester in [10] effectively enhanced energy conversion from low-frequency mechanical vibrations in wearable and human-body environments. Other studies in [11,12] adopted various active power management strategies, including Maximum Power Point Tracking (MPPT), storage regulation, and load adaptation. The reference in [13] introduced a passive MPPT technique using half the open-circuit voltage (VOC/2) to locate the optimal energy-harvesting point without additional power overhead. A more comprehensive overview of EH concepts, power management units, and energy storage integration can be found in [6], which further contextualizes the role of system-level optimization in achieving uninterrupted power supply.

In addition, advancements in integrated circuits and MEMS technologies have enabled the precise harvesting of micro-energies from the environment, as demonstrated by the energy-harvesting MEMS platform in [14]. Multi-source energy harvesting has become a new focus. For example, the architecture in [15] has integrated solar and radioisotope sources, enabling long-term autonomous operation in space and special environments.

However, for RF energy harvesters operating in low-power regimes, these existing strategies often rely on active control circuits, which increase EH system complexity and static power consumption [16,17,18]. This makes it difficult for the EH system to operate reliably in long-term low-power scenarios. Therefore, achieving efficient rectification, low-loss power regulation, and stable output under weak RF input remains an urgent technical challenge.

To address this problem, this work presents the design and implementation of a harmonic-recycling passive RF-energy-harvesting system with integrated power management (HR-P-RFEH), specifically targeted at microwatt-level wireless energy-harvesting applications. The HR-P-RFEH system adopts a dual-stage voltage multiplier architecture. It introduces a microstrip stub-based harmonic suppression and energy-recycling mechanism to improve rectification efficiency and DC output capability. Operating at 915 MHz, the HR-P-RFEH system integrates an impedance matching network, a cascaded diode-based rectifier, and a frequency-selective rejection structure. Under a 0 dBm input, the HR-P-RFEH system achieves a 1.4 V DC output with a peak efficiency of 70.7%, exceeding conventional single-stage designs by over 16.4%.

For the power management of HR-P-RFEH, the ultra-low-power BQ25570 is adopted to boost the rectified voltage to 3.3 V, paired with a 47 mF low-leakage supercapacitor to ensure energy buffering and reliable delivery. Experimental validation shows that the HR-P-RFEH enables continuous powering of sensor nodes under intermittent or microwatt-level RF input, fulfilling the supply requirements of IoT terminals with burst communication.

The major contributions of this work are summarized as follows:

1. A passive dual-stage rectifier architecture integrating harmonic suppression and secondary rectification is proposed to enhance RF-DC efficiency without additional active components.
2. A microstrip stub-based harmonic rejection network is developed to selectively reflect and recycle residual high-frequency energy.
3. A theoretical model of system efficiency is established to quantify the performance gain from energy recycling.
4. A complete low-power energy storage chain is built by combining the BQ25570 and low-leakage supercapacitor, improving voltage regulation and system endurance.

Recent RF energy harvesters often rely on complex strategies, such as active rectifiers or multi-phase topologies to extend the input power range [3,19], dual-band stop networks for harmonic suppression [20], or varactor-based dynamic tuning to improve impedance matching [1]. By contrast, the proposed HR-P-RFEH achieves comparable or superior performance at 0 dBm input using a fully passive, planar, and MEMS-compatible architecture, eliminating the need for additional active control or tuning circuits.

In addition to serving as alternative power supplies, RF energy harvesters have also been increasingly integrated into wireless sensing systems. Prior studies demonstrated their feasibility in diverse applications, such as gesture recognition [21], human activity recognition [22], battery-free wearable platforms [23], and even security-oriented sensing scenarios like mobile app activity monitoring [24]. More recently, REHSense highlighted the potential of fully battery-free wireless sensing enabled solely by RF energy harvesting [25]. These works underline that RF energy harvesting not only supports energy autonomy but can also open new paradigms of sensing and interaction. Nevertheless, for ultra-low-power IoT deployments, achieving consistently high rectification efficiency within a compact, fabrication-friendly architecture remains challenging. This motivates the present work, which introduces a harmonic-recycling passive RF-energy-harvesting system with integrated power management (HR-P-RFEH).

The architecture of the paper is organized as follows: Section 2 describes the detailed circuit design of the proposed HR-P-RFEH system, including the rectifier topology and harmonic suppression mechanism. Section 3 presents the system implementation and experimental validation, with an emphasis on performance evaluation under low-power input conditions. Section 4 concludes the paper and discusses its potential applications in IoT- and MEMS-based platforms.

## 2. Circuit Design

To achieve efficient RF-to-DC conversion under low-power input conditions (0 dBm), this work proposes a dual-stage voltage multiplier rectifier (VMR) topology integrated with a harmonic suppression mechanism in the HR-P-RFEH system. The entire circuit adopts a fully planar microstrip-based implementation, making it compatible with standard MEMS fabrication processes and enabling potential monolithic or system-in-package (SiP) integration. The electromagnetic simulations and circuit-level validations were carried out using Advanced Design System (ADS 2022, Keysight Technologies), which ensured accurate co-simulation of microstrip layouts and nonlinear diode behavior.

The design and validation of the proposed HR-P-RFEH followed a combined simulation–fabrication–measurement workflow. First, the rectifier topology was synthesized at 915 MHz under a 50 Ω source assumption and a target load of 2 kΩ. Nonlinear HSMS-285C diode models were used to account for harmonic generation. All passive components, including stubs and interdigital capacitors, were implemented in planar microstrip on FR4 (H=1.6mm, $\varepsilon_{r}=4.4$, tanδ=0.02). Full-wave EM models (ADS Momentum) were co-simulated with circuit-level harmonic-balance simulations (ADS 2022) to optimize impedance matching and suppression characteristics.

Second, the PCB layout of the prototypes was designed using Altium Designer 2024 and fabricated through photolithography, and measurements were carried out with a calibrated RF source (50 Ω, 915 MHz, −10 to +10 dBm). The rectified output was observed across a 2kΩ load and compared to a single-stage baseline design of identical footprint and diode type. DC voltages were recorded using a high-impedance digital multimeter (DMM), while $S_{11}$ and $S_{21}$ parameters were measured using a Vector Network Analyzer (VNA) after short–open–load–through (SOLT) calibration. Finally, each measurement point was averaged over five repeated experiments, and the rectification efficiency was calculated accordingly. This workflow ensures that the reported efficiency improvements stem directly from the harmonic-recycling mechanism, rather than from differences in device size or measurement conditions.

**Figure 1:**
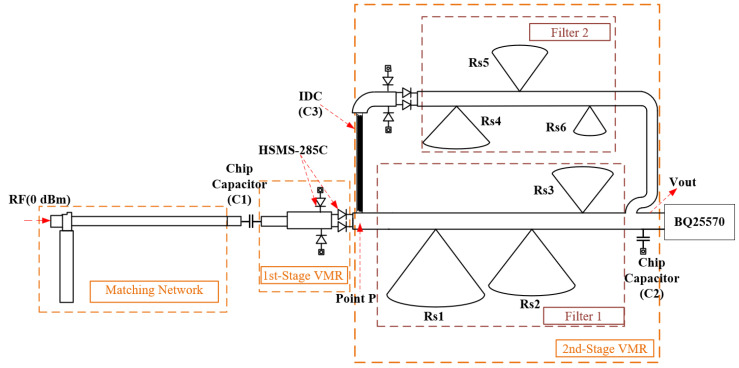
Topology of the HR-P-RFEH system circuit.

Operating at 915 MHz, the HR-P-RFEH system combines a microstrip-matching network, the first-stage voltage multiplier rectifier (1st-Stage VMR), and the second-stage voltage multiplier rectifier (2nd-Stage VMR). Figure 1 illustrates the topology of the HR-P-RFEH system. The RF signal received by the antenna is first passed through the microstrip-matching network. Then, the RF signal is delivered to the 1st-Stage VMR, where it is partially converted into DC. Finally, the residual high-frequency components are selectively redirected into the 2nd-Stage VMR. The 2nd-Stage VMR is integrated with frequency-selective harmonic suppression and the BQ25570-based energy management unit. Through this harmonic suppression path, the HR-P-RFEH system not only enhances rectification efficiency and overall energy conversion but also effectively utilizes residual harmonic energy through harmonic recycling—energy that would otherwise be dissipated or reflected back to the source in conventional rectifiers.

### 2.1. Rectifier Circuit Design

**Figure 2:**
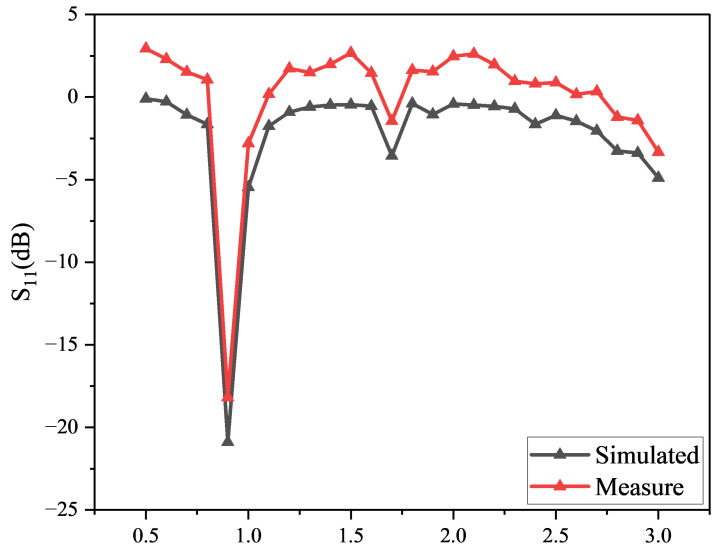
Return loss of the matching network at 915 MHz.

Due to their compact size, light weight, and ease of integration, microstrip lines are widely used as filters in wireless communication and microwave integrated circuits. In the HR-P-RFEH system, both the microstrip-matching network and the 2nd-Stage VMR are implemented using planar microstrip technology compatible with MEMS processes. The microstrip-based matching network enables broadband and stable impedance tuning, effectively addressing the limitations of traditional lumped-element-matching networks. As shown in Figure 2, the simulation results demonstrate that the reflection coefficient $S_{11}$ drops below −40dB at the target frequency, significantly reducing input power loss and improving overall RF-to-DC conversion efficiency. The planar configuration also allows the design to be readily fabricated using standard photolithography and thin-film metallization techniques, enhancing its manufacturability for large-scale integration.

The proposed HR-P-RFEH system adopts a basic voltage multiplier topology in the 1st-Stage VMR, employing HSMS-285C Schottky diodes for preliminary RF-to-DC conversion. Although this stage achieves high conversion efficiency, the nonlinear characteristics of the diode inherently introduce higher-order harmonics into the output [26], causing residual high-frequency energy to leak into the subsequent stages. This phenomenon is primarily attributed to two factors. First, the RF input exhibits a continuous spectral distribution, which cannot be fully absorbed by the rectifier at a single frequency. Second, the nonlinear conduction behavior of the diode generates second-, third-, and higher-order harmonic components. These residual frequencies typically occur at integer multiples of 915 MHz. The rectifier’s response to the input RF signal $v\left(t\right)=v_{0}\cos\left(\omega_{0}t\right)$ can be approximated by a Taylor series expansion, and the output voltage can be expressed as follows:

(1) $$V_{\mathrm{out}}\left(\omega_{0}\right)=G_{0}+\frac{G_{2}v_{0}^{2}}{2}+\left(G_{1}v_{0}+\frac{3G_{3}v_{0}^{3}}{4}\right)\cos\left(\omega_{0}t\right)+\ldots$$

To further improve the rectification efficiency under low-power RF input conditions (0 dBm), the HR-P-RFEH system introduces a 2nd-Stage VMR in a fully planar configuration compatible with MEMS fabrication processes. This stage processes high-frequency components that are not absorbed in the 1st-Stage VMR. The 2nd-Stage VMR forms a frequency-selective, band-stop structure using a microstrip stub to selectively reflect residual harmonics at the fundamental frequency (915 MHz) and its multiples. This achieves simultaneous “suppression and recycling”, mitigating common issues in traditional rectifier designs, such as device non-idealities, harmonic interference, and reflection loss [5], thereby improving DC output stability. As further validated in [20], dual-band stop networks have been proven effective in enhancing rectification efficiency and improving DC output stability through harmonic energy management. In the practical design, the suppression frequency and bandwidth are directly determined by the dimensions and positioning of the microstrip stub. Based on an equivalent transmission line model, the input impedance of the open-circuited stub is derived and used to construct a band-stop structure in the frequency domain. Inspired by the polarization- and angle-insensitive energy-harvesting network proposed in [27], this work adopts a similar microstrip-based frequency-selective design, which demonstrates robust frequency locking and is insensitive to incident direction.

When the nonlinear conduction of the Schottky diode generates higher-order harmonics (mainly at $2f_{0}$, $3f_{0},\ldots$), these residual components appear at the output of the 1st-Stage VMR. Without suppression, they would propagate either to the load or back to the source, causing wasted energy and mismatch loss. The open-circuited microstrip stub is designed to resonate at $f_{0}=915$ MHz and selected harmonics.

At resonance, the stub presents very low impedance (see Equation (6)), effectively short-circuiting the unwanted harmonic currents and reflecting them away from the load path. Instead of being dissipated, this reflected RF energy is redirected through the interdigital capacitor $C_{3}$ into the 2nd-Stage VMR, where it undergoes secondary rectification. In this way, the stub simultaneously performs harmonic suppression (by preventing leakage of high-frequency components to the load) and harmonic recycling (by steering the residual RF energy into a dedicated rectification path). This dual role distinguishes the proposed design from conventional stubs, which usually only suppress but do not recycle harmonics. As a result, both DC stability and overall RF-to-DC efficiency are significantly improved.

This design, while enhancing RF-to-DC efficiency, is compact, lightweight, and suitable for monolithic integration in chip-scale or SiP platforms, significantly reducing high-frequency energy loss via localized filtering, reflection, and rectification [28].

**Figure 3:**
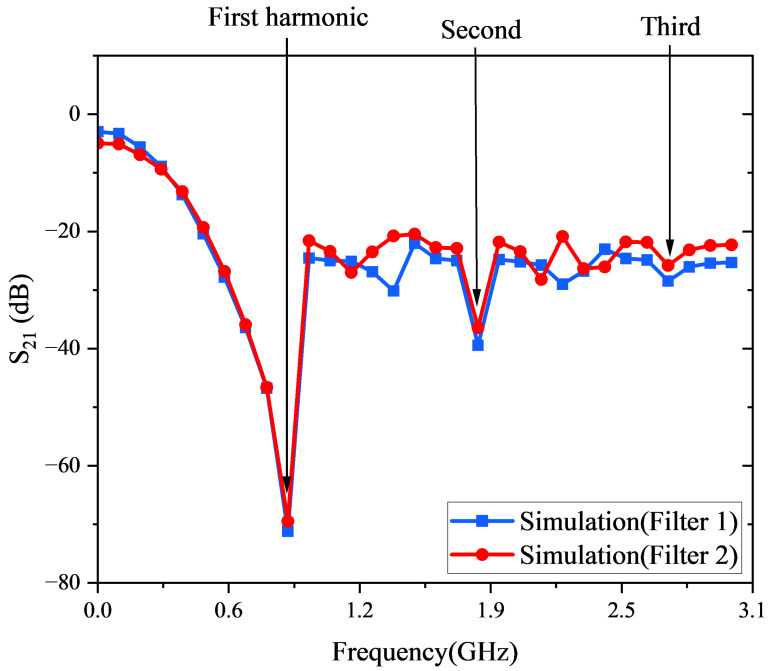
S21 response of the dual harmonic suppression filters.

To evaluate the harmonic suppression behavior, the S21 parameter of the 2nd-Stage VMR is analyzed. As shown in Figure 3, the stage includes two filters—Filter1 and Filter2—each targeting specific harmonic ranges. Filter1 suppresses the fundamental, as well as the second and third, harmonics, redirecting residual energy through the DC-blocking capacitor $C_{3}$ into Filter2. Filter2 further attenuates any remaining harmonic components, ensuring a smoother and more stable DC output. As a result, the 2nd-Stage VMR produces prominent suppression bands in the S21 transmission response. The rejection depths and bandwidths are determined by the microstrip geometry, which is fully compatible with standard MEMS lithographic and metallization techniques. In practice, fabrication tolerances in stub length and microstrip width may cause slight resonance shifts and matching degradation. Nevertheless, tolerance analysis with ±5% dimensional variations showed less than 3% deviation in rectification efficiency, confirming robustness against typical PCB and MEMS lithography errors. Moreover, since the stubs operate as quarter-wave resonators directly integrated at the rectifier input, their suppression effect depends primarily on guided wavelength rather than the polarization or incident angle of the incoming RF field. This ensures that the harmonic recycling mechanism remains effective under both fabrication and excitation variations, supporting stable performance in real-world deployment scenarios.

To analyze the frequency-selective characteristics of the microstrip stub, transmission line theory is applied to derive its input impedance. Let $Z_{0}$ denote the characteristic impedance and θ=βl, where β is the propagation constant, and *l* is the physical length of the stub. The input impedance of the open-circuited stub is given by the following:

(2) $$Z_{\mathrm{in}}\left(\omega \right)=jZ_{0}\tan\left(\theta \right)$$

The microstrip stub is designed to operate as a frequency-selective shunt path. At resonance, the quarter-wave open-circuited stub transforms into a short circuit, providing very low shunt impedance at the design frequency. This allows for residual harmonic currents to be selectively diverted into the 2nd-Stage VMR for recycling. Away from resonance, however, the stub exhibits a very high impedance, leaving the main rectification path unaffected. Moreover, the DC-blocking interdigital capacitor $C_{3}$ ensures that only high-frequency residual components are coupled into the recycling branch, preventing any leakage of the stored DC energy. As a result, the 2nd-Stage VMR achieves harmonic recycling without increasing insertion loss or static power consumption.

The effective dielectric constant of the microstrip line is estimated using the following standard approximation:

(3) $$\varepsilon_{\mathrm{eff}}=\frac{\varepsilon_{r}+1}{2}+\frac{\varepsilon_{r}-1}{2}\cdot \frac{1}{\sqrt{1+12H/W}}$$

where $\varepsilon_{r}$ is the substrate relative permittivity, *H* is the substrate thickness, and *W* is the microstrip width.

The guided wavelength is then given by

(4) $$\lambda_{g}=\frac{c}{f\sqrt{\varepsilon_{\mathrm{eff}}}}$$

where *c* is the speed of light in free space, and *f* is the operating frequency.

Based on this, the stub length is chosen close to a quarter guided wavelength,

(5) $$L\approx \frac{\lambda_{g}}{4}=\frac{c}{4f\sqrt{\varepsilon_{\mathrm{eff}}}}$$

which corresponds to the resonance relation

(6) $$f_{0}=\frac{c}{4L\sqrt{\varepsilon_{\mathrm{eff}}}}$$

For the FR4 substrate used in this work ($\varepsilon_{r}=4.4$, H=1.6 mm, W≈3 mm), Equation (3) yields $\varepsilon_{\mathrm{eff}}\approx 3.9$. At $f_{0}=915$ MHz, the guided wavelength is $\lambda_{g}\approx 124$ mm, leading to a stub length of approximately L≈31 mm. The stubs are connected in parallel with the input of the 2nd-Stage VMR, at the junction where harmonic currents accumulate, to maximize suppression at the fundamental and low-order harmonics while simultaneously recycling the reflected energy.

Since all dimensions are defined in planar geometry, they can be directly fabricated using MEMS-compatible thin-film deposition and etching processes, facilitating monolithic integration with other RF front-end circuitry.

If the residual high-frequency energy from the 1st-Stage VMR is directly transmitted to the load or other circuit sections, it cannot be efficiently converted to DC due to frequency mismatch, resulting in energy waste. To address this problem, an interdigital capacitor $C_{3}$ is placed at point *P* to block the DC component while constructing a return path for the high-frequency component. The isolated high-frequency energy is redirected through the coupled branch into the second-stage voltage multiplier rectifier, enabling a “reflection–re-rectification” mechanism for energy recycling. The interdigital capacitor structure is also planar and scalable, supporting direct integration into MEMS or low-temperature co-fired ceramic (LTCC) processes for compact energy-harvesting front ends.

### 2.2. Power Management Circuit Design

In most rectifier modules, the rectified DC output is approximately 1 V, which is insufficient to directly drive typical IoT loads such as wireless sensor nodes. To enable efficient accumulation and regulated supply of such weak harvested energy, a low-power and highly adaptive power management unit is required [19]. In this work, the Texas Instruments BQ25570 chip is adopted as the core of the power management module, based on the following design considerations:

1. BQ25570 supports a cold-start threshold as low as 600 mV, and after startup, it can continue operating with input voltages down to 100 mV [29]. This makes it particularly well-suited for microwatt-level RF energy input.
2. Given that the proposed system uses a fully passive RF rectifier without any external microcontroller, the periodic-sampling MPPT strategy of the BQ25570 allows for maximum power point tracking without introducing additional active power consumption.
3. As the system replaces traditional batteries with supercapacitors, the BQ25570 integrates supercapacitor charge/discharge management, voltage threshold control, and LDO regulation, thereby providing efficient energy accumulation and a stable voltage supply to the back-end MCU [19].

**Figure 4:**
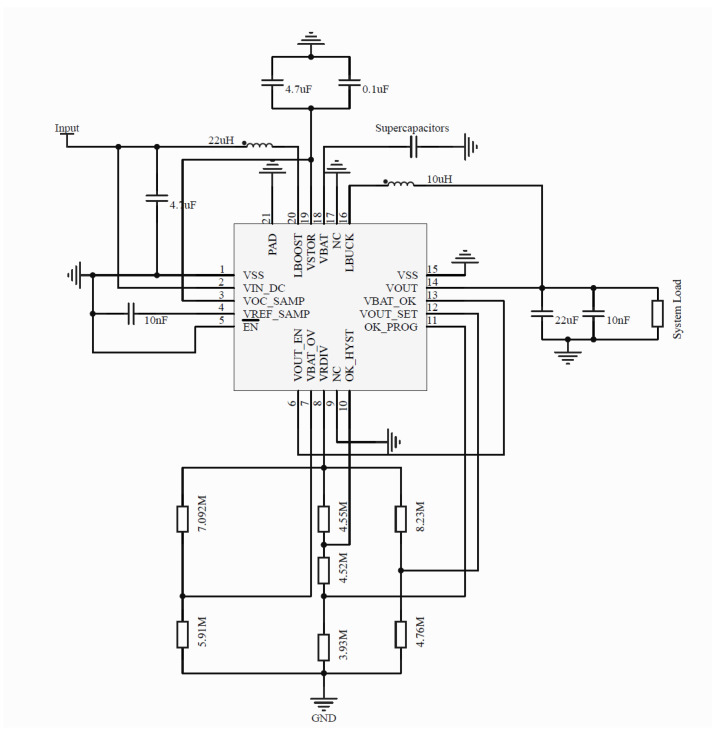
BQ25570 power management circuit.

Figure 4 shows the circuit schematic of the energy management module. The rectifier output is connected to the input of the BQ25570, which can be modeled as a DC voltage source $V_{\mathrm{in}}$ in series with an input resistance $R_{s}$, expressed as follows:

(7) $$V_{\mathrm{source}}=V_{\mathrm{in}}-I_{\mathrm{in}}R_{s}$$

The BQ25570 not only boosts the harvested voltage but also regulates and stores the energy with high efficiency. In the sub-milliwatt regime relevant to this work, the BQ25570 typically achieves 80–90% conversion efficiency, ensuring that most of the energy collected by the rectifier (70.7% RF-to-DC efficiency at 0 dBm) is preserved during regulation and storage. Its built-in MPPT and energy-buffering functions further mitigate the impact of fluctuating RF input, allowing for the overall system to maintain stable delivery with only modest efficiency penalties compared to the rectifier output. The regulated output of 3.3 V is buffered by a supercapacitor and used to drive a sensor node or other low-power controllers. All interconnections are realized on a planar PCB/microstrip platform, making the module compatible with MEMS-based integration or hybrid system-in-package assembly. This design eliminates the need for additional active control circuitry, significantly simplifying the overall system while ensuring manufacturability and scalability for compact, low-power IoT applications.

## 3. System Implementation

This section presents the experimental validation and system-level implementation of the proposed HR-P-RFEH system, emphasizing its planar, MEMS-compatible architecture. The prototype integrates the dual-stage voltage multiplier rectifier (VMR) and harmonic suppression network on a compact FR4 microstrip substrate, with all passive elements designed in planar form to support future transfer to silicon-based or LTCC (Low-Temperature Co-Fired Ceramic) platforms. The system evaluation covers the construction and testing of the VMR, performance assessment under various RF input levels, and integration of the BQ25570-based power management unit. Design considerations for energy storage and leakage mitigation are also discussed, highlighting the feasibility of embedding the entire energy harvesting and regulation chain into a miniaturized, mass-producible module. In addition, it is worth noting the trade-offs when extending the rectifier architecture beyond this design. While adding more voltage multiplier stages can increase the DC output voltage, it inevitably introduces additional diode drops and parasitic capacitances, which raise the input threshold and reduce efficiency under low-power excitation. Likewise, adopting more complex filter configurations (such as multi-band or hybrid resonators) can further suppress harmonics, but they increase circuit size and fabrication complexity, potentially compromising MEMS compatibility. For these reasons, the proposed dual-stage configuration with a single stub-based harmonic recycling network represents a balanced choice, achieving substantial efficiency gains while maintaining compactness and fabrication simplicity.

### 3.1. Implementation and Evaluation of VMR

To evaluate the rectification performance of the VMR, both simulation and experimental validation were conducted under a 915 MHz RF input. The experimental platform employed a standard 50 Ω RF signal source with an output power range from −10dBm to +10dBm, and the VMR was connected to a 2 kΩ resistive load. The dual-stage structure allows the first stage to perform primary RF-to-DC conversion, while the second stage captures and re-rectifies residual high-frequency energy, thereby improving overall energy utilization without requiring discrete, non-planar components.

The theoretical gain from the secondary energy recovery mechanism can be described by the following equation:

(8) $$\eta_{\mathrm{total}}=\eta_{1}+k\left(1-\eta_{1}\right)\eta_{2}$$

where $\eta_{1}$ is the efficiency of the 1st-Stage VMR, *k* is the proportion of residual high-frequency energy, and $\eta_{2}$ is the efficiency of the 2nd-Stage VMR. The second term in Equation (8) represents the additional contribution from recycled harmonic energy. Because the entire harvesting path is implemented with planar microstrip and surface-mounted diodes, this architecture is inherently compatible with MEMS and thin-film integration for compact RF–DC converters.

**Figure 5:**
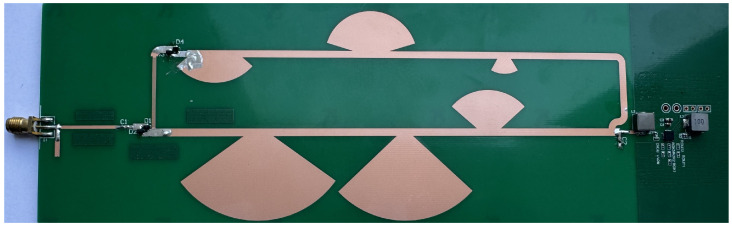
Photograph of the fabricated planar HR-P-RFEH prototype.

The HR-P-RFEH system was implemented on an FR4 substrate. Figure 5 shows the fabricated prototype. All transmission lines, resonant stubs, and interdigital capacitors are realized in a planar layout, enabling straightforward scaling to wafer-level fabrication. Electromagnetic simulations in ADS Momentum were used to co-optimize geometry and impedance, ensuring minimal mismatch and precise frequency alignment. This design methodology—simulation-driven, planar, and photolithography-ready—allows for rapid translation from PCB prototyping to MEMS-compatible microfabrication.

**Figure 6:**
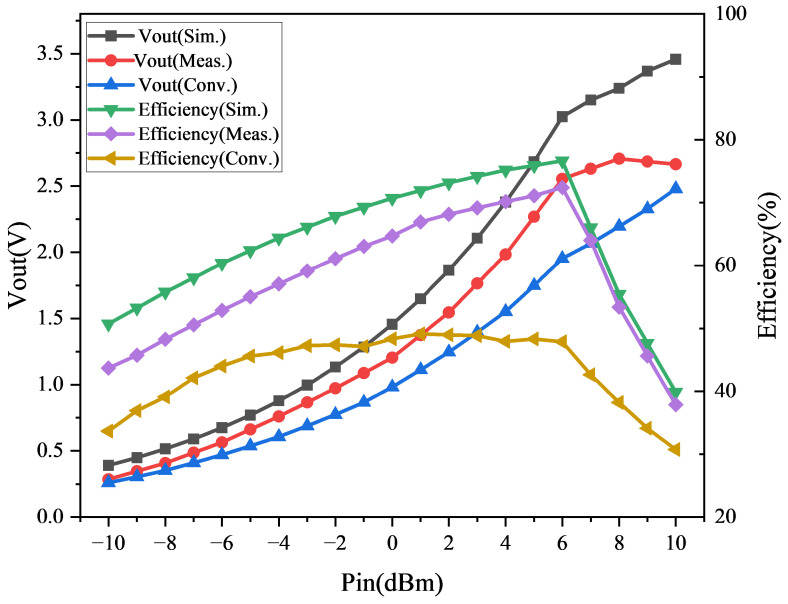
Measured rectification efficiency versus input RF power.

Figure 6 compares the measured and simulated DC output voltage and rectification efficiency for the proposed HR-P-RFEH and a conventional single-stage rectifier. At 0 dBm input and 915 MHz, the proposed system delivers a stable DC output of approximately 1.4 V with negligible ripple. This corresponds to a rectification efficiency of 70.7%, an improvement of 16.4% over the baseline design. The 22.5% increase in output voltage and 16.4% improvement in efficiency at 0 dBm confirm that the harmonic recycling path effectively redirects residual harmonic energy into useful DC power, demonstrating its practical contribution to overall energy utilization.

Beyond this operating point, the rectification efficiency shows a clear dependence on input power. At low input levels (−10 to −6 dBm), the efficiency remains around 50–60% due to the diode threshold and startup-related losses. As the input increases from −4 to 0 dBm, the efficiency rises steeply, reaching 70.7% at 0 dBm. Between 0 and +6 dBm, the efficiency continues to increase and saturates near 78–80%, indicating that the rectifier operates close to its optimum without requiring active bias or control. At higher inputs (8–10 dBm), the efficiency gradually rolls off, which is consistent with increased conduction and parasitic losses at elevated drive levels. This behavior explicitly indicates that the rectifier reaches a saturation point near +6 dBm, beyond which additional RF power no longer improves efficiency and may even reduce it.

It is important to note that this measured behavior across −10 to +10 dBm effectively emulates the variability of real-world RF environments, where incident power fluctuates due to multipath fading, antenna orientation, and temporal changes in ambient RF traffic. The HR-P-RFEH maintains robust rectification efficiency across this dynamic range, highlighting its suitability for practical deployment in low-power IoT and MEMS scenarios. Furthermore, integration with the BQ25570-based power management unit ensures stable energy storage and regulated delivery, further mitigating the impact of input power variability. While the fully planar design is inherently compatible with MEMS and wafer-level fabrication, scaling presents challenges such as tighter tolerances for stub length and interdigital capacitor gaps, and the need to minimize substrate losses at small geometries. Nevertheless, the absence of active devices and the use of standard planar processes make HR-P-RFEH well suited for large-scale manufacturing and monolithic integration.

The rectification efficiency is calculated as follows:

(9) $$\eta =\frac{V_{\mathrm{out}}^{2}}{R_{L}\cdot P_{\mathrm{in}}}$$

where $R_{L}$ is the load resistance, and $V_{\mathrm{out}}$ is the DC output voltage. The observed saturation of efficiency at higher input levels suggests that the architecture reaches an optimal operating point without requiring active bias or control, which further supports fully passive, microfabrication-friendly implementation.

### 3.2. Power Management and Energy Output Validation

To ensure stable power delivery to downstream loads during intermittent RF input, a 47 mF low-leakage supercapacitor is connected to the output of the BQ25570 unit. The minimum required capacitance $C_{\min}$ can be estimated from the following equation:

(10) $$C_{\min}=\frac{2E}{V_{\max}^{2}-V_{\min}^{2}}$$

For an energy requirement E≤3mJ and voltage range 2.0–3.3 V, $C_{\min}\approx 770\;\mu F$. Therefore, the 47 mF value was selected to provide a wide safety margin beyond the calculated $C_{\min}\approx 770\;\mu F$. This large capacitance ensures smoother charging profiles and longer discharge intervals, thereby improving voltage stability during intermittent RF input. At the same time, the oversized capacitance slightly increases the charging time, but it significantly enhances the number of usable charge/discharge cycles by reducing voltage fluctuations and avoiding deep discharge stress on the storage element.

The charging process of the supercapacitor follows the equation below:

(11) $$V_{\mathrm{cap}}\left(t\right)=V_{\mathrm{target}}\left(1-e^{-\frac{t}{R_{\mathrm{in}}C}}\right)$$

where $V_{\mathrm{target}}$ is the target voltage. The discharging behavior is given by the equation below:

(12) $$V_{\mathrm{cap}}\left(t\right)=V_{\mathrm{start}}\cdot e^{-\frac{t}{R_{L}C}}$$

All energy storage elements are in planar packaging, and the supercapacitor can be implemented as a thin-film device in future MEMS-based modules.

Leakage energy loss is modeled by the following equation:

(13) $$E_{\mathrm{leak}}=I_{\mathrm{leak}}\cdot V_{\mathrm{avg}}\cdot t_{\mathrm{idle}}$$

where $V_{\mathrm{avg}}=\left(V_{\max}+V_{\min}\right)/2$. A supercapacitor with $I_{\mathrm{leak}}<1\;\mu A$ was selected to minimize standby loss. The BQ25570’s undervoltage lockout was configured at 2.6 V to further protect energy retention. Under a 0 dBm RF input, the integrated rectifier–PMU chain delivers a regulated 3.3 V to the load, validating the system’s ability to operate as a compact, battery-free power source for MEMS-scale wireless sensor nodes.

## 4. Conclusions

This work presents a fully passive, dual-stage harmonic-recycling RF-energy-harvesting system with integrated power management, referred to as HR-P-RFEH. The design adopts a planar microstrip architecture, inherently compatible with MEMS fabrication processes and suitable for wafer-level or SiP integration. The proposed topology incorporates a microstrip-stub-based harmonic suppression and recycling mechanism between two voltage multiplier rectifiers (VMRs), enabling efficient utilization of residual harmonic energy.

Experimental results at 915 MHz under a low input power of 0 dBm and a 2 kΩ load demonstrate a stable DC output of 1.4 V and a peak rectification efficiency of 70.7%. Compared with a conventional single-stage rectifier, the HR-P-RFEH achieves a 22.5% increase in output voltage and a 16.4% improvement in efficiency. The rectified output is further boosted and regulated to 3.3 V via an ultra-low-power BQ25570 power management unit, with energy buffered in a 47 mF low-leakage supercapacitor to provide stable operation even under intermittent RF input.

In comparison with the state of the art, the proposed harmonic-recycling, fully passive architecture demonstrates that high rectification efficiency can be achieved at 0dBm input without active bias, control loops, or non-planar components. By converting otherwise wasted harmonic content into useful DC through a selective suppression–redirection path, HR-P-RFEH raises $V_{\mathrm{OUT}}$ by 22.5% and efficiency by 16.4% over an equal-footprint baseline, while remaining MEMS- and LTCC-ready. This combination of efficiency, simplicity, and manufacturability makes HR-P-RFEH a compelling building block for battery-free IoT and MEMS nodes that must operate from intermittent, microwatt-level RF fields.

Looking forward, the HR-P-RFEH concept can be further extended in two main directions. First, optimizing the harmonic recycling path or employing multi-resonant stub networks could increase the fraction of residual energy converted into useful DC power, pushing efficiency beyond the current limits. Second, extending the design to cover multiple frequency bands or incorporating ultra-low-power adaptive tuning may enhance robustness under diverse and fluctuating RF environments. These prospective improvements highlight the adaptability of the HR-P-RFEH architecture and its promise for next-generation energy-autonomous microsystems.

## Data Availability

No new data were created or analyzed in this study.

## Abbreviations

The following abbreviations are used in this manuscript:

IoT	Internet of Things
RF	Radio Frequency
HR-P-RFEH	Harmonic-Recycling Passive RF Energy Harvester
MEMS	Micro-Electro-Mechanical System
VMR	Voltage Multiplier Rectifier
PCB	Printed Circuit Board
DC	Direct Current
LTCC	Low-Temperature Co-Fired Ceramic
EH	Energy Harvesting
MPPT	Maximum Power Point Tracking
VOC	Open-Circuit Voltage
SiP	System-in-Package
ADS	Advanced Design System
FR4	Flame Retardant 4 (PCB substrate)
EM	Electromagnetic
DMM	Digital Multimeter
VNA	Vector Network Analyzer
SOLT	Short-Open-Load-Thru (VNA calibration)
S11	Input Reflection Coefficient
S21	Forward Transmission Coefficient
LDO	Low Dropout Regulator
MCU	Microcontroller Unit
PMU	Power Management Unit
